# Regenerative Synergy in Facial Rejuvenation: A Pilot Clinical Study of Polydioxanone Microspheres (Ultra V® UltraCol 200), Organic Silicon, and Adipostructuring

**DOI:** 10.7759/cureus.104912

**Published:** 2026-03-09

**Authors:** Marta Amin, Victor Mercado, Gladys Velazco, Valentina Corvalan, Han Jin Kwon

**Affiliations:** 1 Dentistry, Instituto Chileno de Rejuvenecimiento y Optimización en Medicina Estética, Viña del Mar, CHL; 2 Otolaryngology, Instituto de Neurorrehabilitación y Equilibrio, Viña del Mar, CHL; 3 Regenerative and Aesthetic Medicine, Centro Latinoamericano de Entrenamiento Médico e Investigación, Bogotá, COL; 4 Medicine and Anatomy, La Universidad de los Andes, Bogotá, VEN; 5 Aesthetic Medicine, Clínica Roma, Santiago, CHL; 6 Dermatology, UltraV Co. Ltd. Research and Development Center and Dermaster Clinic Network, Seoul, KOR

**Keywords:** adipostructuring, liquid polydioxanone, neocollagenesis, organic silicon, synergy, ultracol 200 ultrav

## Abstract

Background

Dermal fillers used with minimally invasive techniques have currently achieved an important role in regenerative medicine and in alleviating skin damage over the years.

Objectives

To evaluate clinical results after the use of the Polydioxanone Collagen Biostimulator Ultra V® UltraCol 200 (Ultracol), commonly referred to as PDO microspheres, with the addition of organic silicon to enhance Ultracol biostimulation, using the adipostructuring technique in a group of older patients who consulted to improve the quality of their skin.

Materials and methods

The study included 20 patients (3 men and 17 women), aged 40 to 80 years. We used Ultracol plus organic silicon. Injections were performed using the facial adipostructuring technique, with a 22-gauge, 50-mm cannula, injecting 2.5 mL per hemiface in a single session. We evaluated results at 10 days and 2 months after the procedure using photography and parameters selected by a blinded evaluator, along with a satisfaction survey.

Results

Clinical evaluation demonstrated noticeable improvement in facial tridimensionality, with enhanced volumetric projection and contour definition. A reduction in nasolabial fold depth was observed, along with improved vertical positioning of the malar region. Additionally, better definition of the infraorbital area was noted, accompanied by a subtle increase in supraperiosteal volume. Overall, these changes were considered favorable outcomes in this treated older patient group.

Conclusion

Ultracol plus organic silicon, using the facial adipostructuring technique in a group of older patients, can be considered a suitable and safe therapeutic alternative.

## Introduction

Facial skin aging is characterized by a progressive loss of firmness, elasticity, and hydration, accompanied by the development of wrinkles, skin laxity, and a fatigued appearance [1-3]. The factors involved in this process are both intrinsic and extrinsic; however, UV radiation is considered the most significant external contributor [4]. Current biostimulators vary in composition, properties, and application protocols, and, overall, favorable clinical outcomes have been reported with agents such as poly-L-lactic acid (PLLA), polycaprolactone (PCL), calcium hydroxyapatite, polyglycolic acid (PGA), and polydioxanone (PDO). These agents have demonstrated efficacy in mitigating the visible effects of skin aging [5].

Liquid PDO Ultra V® UltraCol 200 (Ultracol) is a crystalline biodegradable polymer composed of repeating ether-ester units. It undergoes hydrolytic degradation and is primarily eliminated through renal excretion, with residual byproducts being metabolized and exhaled as CO₂. The degradation process is typically completed within six months [6-7]. The aesthetic application of Ultracol began in 2020. This biostimulator consists of PDO microspheres characterized by a smooth surface and uniform spherical morphology. It is supplied as a lyophilized powder in 100- and 200-mg vials and is reconstituted with sterile water for injection prior to administration, with the aim of stimulating neocollagenesis [8].

Published studies indicate that liquid PDO, in addition to its biocompatibility, stimulates the production of type I and type III collagen [8]. A key characteristic of Ultracol is its rapid biodegradation and controlled inflammatory response, features comparable to those of other collagen biostimulators [9]. These properties make it particularly suitable for patients seeking moderate, minimally invasive facial biostimulation with transient effects and a favorable safety profile [10]. In addition, Organic Silicon has demonstrated regenerative properties and may enhance the biostimulatory effects of PDO-based materials [11]. Facial adipostructuring (FA) is a minimally invasive technique designed to reorganize facial adipose compartments through vector-based mechanical stimulation, and this approach was employed in the present study [12].

## Materials and methods

This pilot, observational, and descriptive clinical study included 20 patients (17 females and 3 males) with a mean age of 63.3 years (range: 40-80 years). Patients with autoimmune diseases, collagen disorders, pregnancy, active malignancy, ongoing chemotherapy or immunotherapy, infectious conditions, uncontrolled diabetes, or inability to comply with follow-up were excluded.

All participants sought improvement in facial skin quality and requested subtle aesthetic enhancement without volumetric overcorrection. Prior to treatment, all patients received detailed information regarding the products and injection technique, and provided written informed consent.

The biostimulator used was Ultracol combined with Organic Silicon (Si). All procedures were performed by a single aesthetic medicine specialist using the FA technique. Treatments were conducted in a private clinic authorized by the Ministry of Health of Chile (Resolution No. 1743).

**Figure 1:**
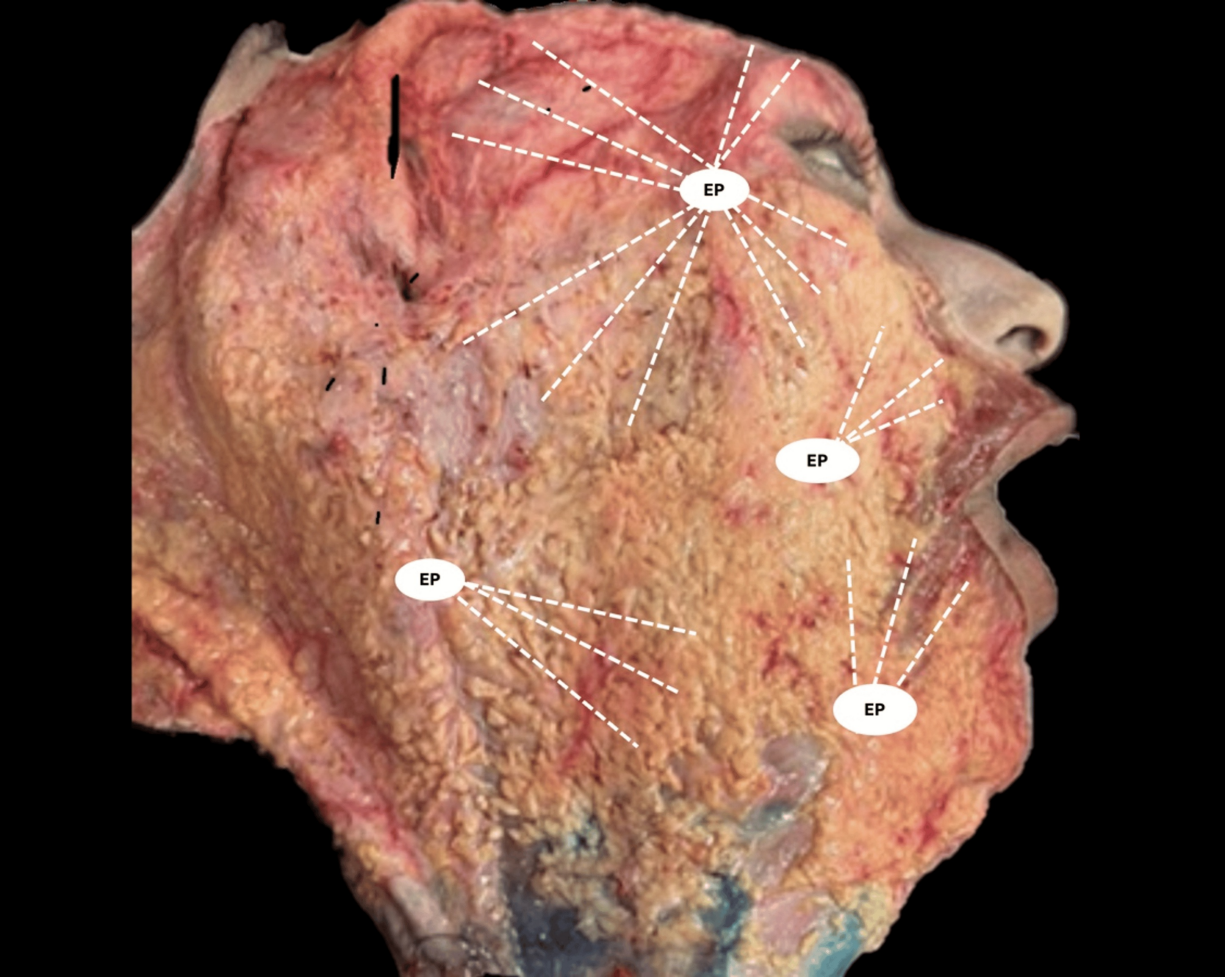
Superficial facial dissection illustrating the vector system used. Schematic representation of the vector distribution applied to the supraorbital, temporal, supraparotid, gonial, and jowl regions during facial adipostructuring. Image reproduced with permission from author Velazco G (Centro Latinoamericano de Entrenamiento Médico e Investigación (CLEMI), Bogotá, Colombia (2025)). EP: Entry point.

The treated regions included the temporal, supraorbital, supraparotid, nasolabial fold, jowl, and mandibular border areas. A 22-gauge, 50-mm cannula was used. Ultracol (one vial) was reconstituted with 3 mL of sterile water for injection, combined with 1 mL of lidocaine with epinephrine and 1 mL of Si. A total of 2.5 mL was injected per hemiface, distributed as 0.25 mL per vector in the previously described areas (Figure 1).

Patients were followed up at 10 days and 3 months post-procedure. Standardized photographs were obtained at each visit using the same camera and imaging protocol. Patient confidentiality was maintained in accordance with the principles of the Declaration of Helsinki.

Outcome assessment was performed by a blinded evaluator, a specialist in aesthetic medicine, who had access exclusively to the photographic records. Image analysis was conducted using FotoDoc Pro software. At the three-month follow-up, patients completed the validated Global Aesthetic Improvement Scale (GAIS), a 5-point subjective assessment tool widely used in aesthetic clinical studies to evaluate overall treatment outcomes.

This study was reviewed and approved by the Research Ethics Committee of Faculdade Centro-Oeste Paulista (FACOP), Brazil (Approval No. FACOP-IRB-0017-2026). All participants provided written informed consent for treatment and publication. The study was conducted in accordance with the Declaration of Helsinki and Good Clinical Practice guidelines.

## Results

**Figure 2:**
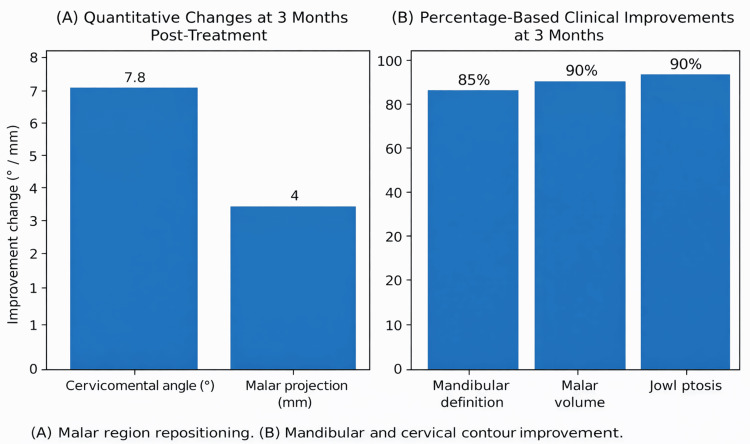
Structural changes observed at 3 months post-treatment. (A) Quantitative anthropometric changes, including a reduction in the cervicomental angle (7.8°) and an increase in malar projection (4 mm). (B) Percentage-based clinical improvement rates in mandibular definition (85%), malar volume (90%), and jowl ptosis (90%).

The blinded evaluator performed the facial assessment using the FotoDoc Pro application. The anatomical parameters analyzed included the cervicomental angle, mandibular definition, malar volume, and jowl ptosis. Changes were determined through comparative photographic analysis. The quantitative and percentage-based outcomes are presented in Table 1 and Figures 2A-2B.

**Table 1:** Summary of clinical parameters and observed post-treatment changes, expressed in degrees, millimeters, or percentages, according to the variable analyzed.

Parameter	Observed Change
Cervicomental angle	Increased (~5-10°)
Malar projection	Increased (~3-5 mm)
Mandibular border definition	Markedly improved
Malar volume	Restored
Jowl ptosis	Reduced (90% of cases)

**Figure 3:**
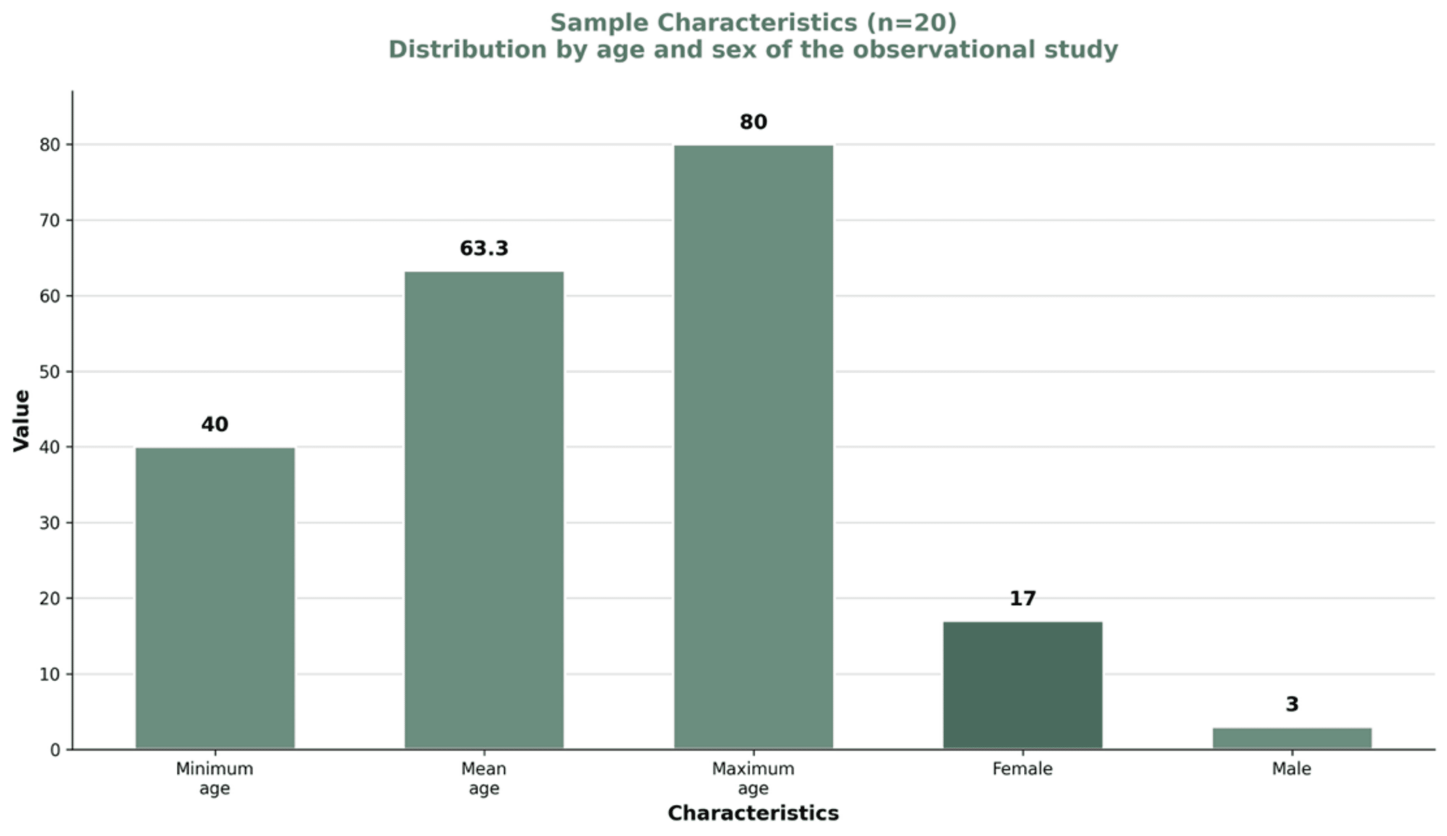
Distribution of patients by age and sex. The mean age was 63.3 years, with a minimum age of 40 years and a maximum age of 80 years. The sample included 17 female and 3 male participants.

The distribution of patients by age and sex is presented in Figure 3. The mean age was 63.3 years (range: 40-80 years). The sample included 17 female and 3 male participants.

**Figure 4:**
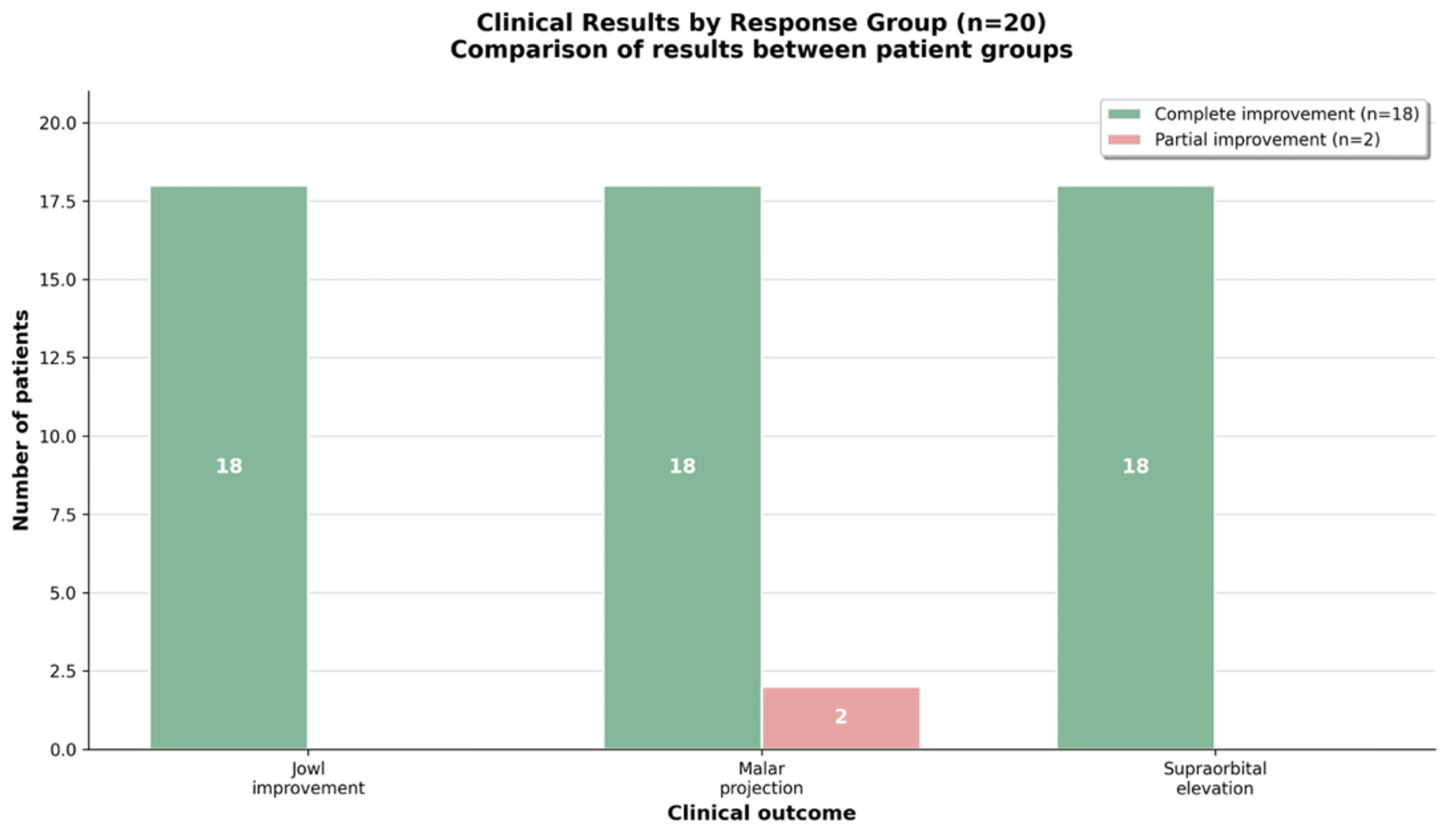
Clinical outcomes by response group. Eighteen patients (90%) achieved complete improvement in jowl definition, malar projection, and supraorbital elevation. Two patients (10%) achieved partial improvement, limited to malar projection.

Clinical outcomes by response group are presented in Figure 4 and Table 2. Eighteen patients (90%) demonstrated complete improvement in jowl definition, malar projection, and supraorbital elevation. Two patients (10%) demonstrated partial improvement, limited to malar projection.

**Table 2:** Distribution of clinical outcomes by response group (n = 20). Eighteen patients demonstrated complete improvement across all evaluated parameters, while two patients demonstrated improvement limited to malar projection.

Parameter	Group 1: Complete Improvement (n = 18)	Group 2: Partial Improvement (n = 2)	Total
Jowl improvement	18 (100%)	0 (0%)	18 (90%)
Malar projection	18 (100%)	2 (100%)	20 (100%)
Supraorbital elevation	18 (100%)	0 (0%)	18 (90%)

Based on the results obtained in this predominantly older adult cohort and the changes observed at the three-month follow-up, the procedure appears to contribute to a more 3D facial profile, with a noticeable reduction in nasolabial fold depth. Improved vertical positioning of the malar region was observed. At the periorbital level, a discrete increase in supraorbital volume was noted, enhancing the definition of the infraorbital region.

These changes may be associated with improved restoration of facial proportions, a more balanced distribution across the facial thirds, enhanced facial harmony, and smoother transitions between anatomical regions.

These findings are consistent with the observation that 18 of 20 patients achieved jowl reduction, despite a mean age of 63.3 years.

**Figure 5:**
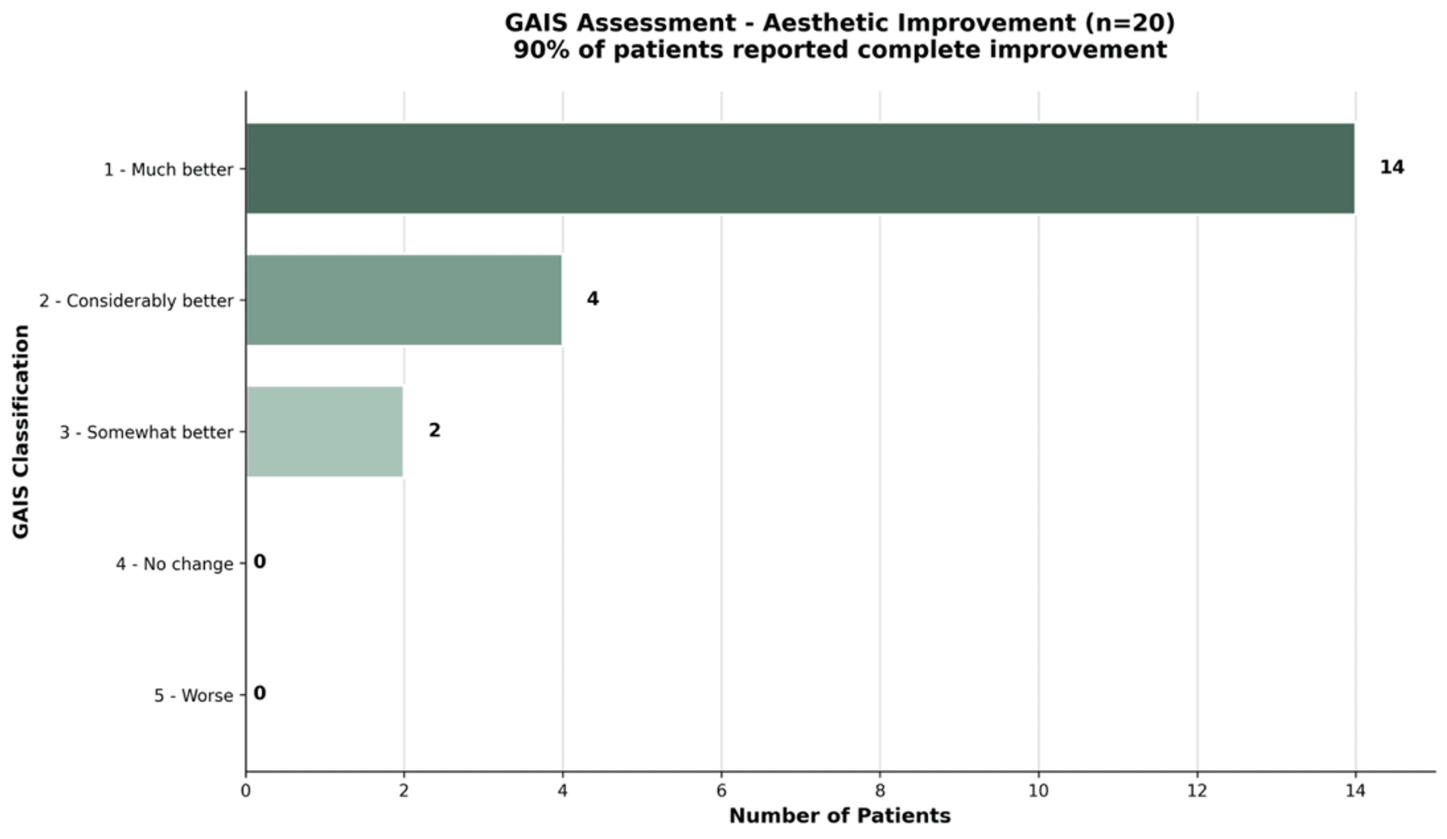
Analysis of aesthetic improvement according to the GAIS scale. Fourteen patients were classified as ‘much better,’ four as ‘considerably better,’ and two as ‘somewhat better.’ No patients reported ‘no change’ or ‘worse’ outcomes at the three-month follow-up. GAIS: Global Aesthetic Improvement Scale.

Regarding the GAIS, 14 of the 20 patients reported great improvement, 4 reported considerable improvement, and 2 reported some improvement, reflecting a high level of patient-perceived satisfaction (Figure 5 and Table 3).

**Table 3:** Distribution of GAIS scores at 3 months (n = 20). Fourteen patients (70%) were classified as ‘very much improved,’ four (20%) as ‘much improved,’ and two (10%) as ‘improved.’ No patients were classified as ‘no change’ or ‘worse.’ GAIS: Global Aesthetic Improvement Scale.

GAIS Scale	Aesthetic Improvement	Patients (n)	Percent
1	Very Much Improved	14	70%
2	Much Improved	4	20%
3	Improved	2	10%
4	No Change	0	0%
5	Worse	0	0%

Clinical interpretation

**Figure 6:**
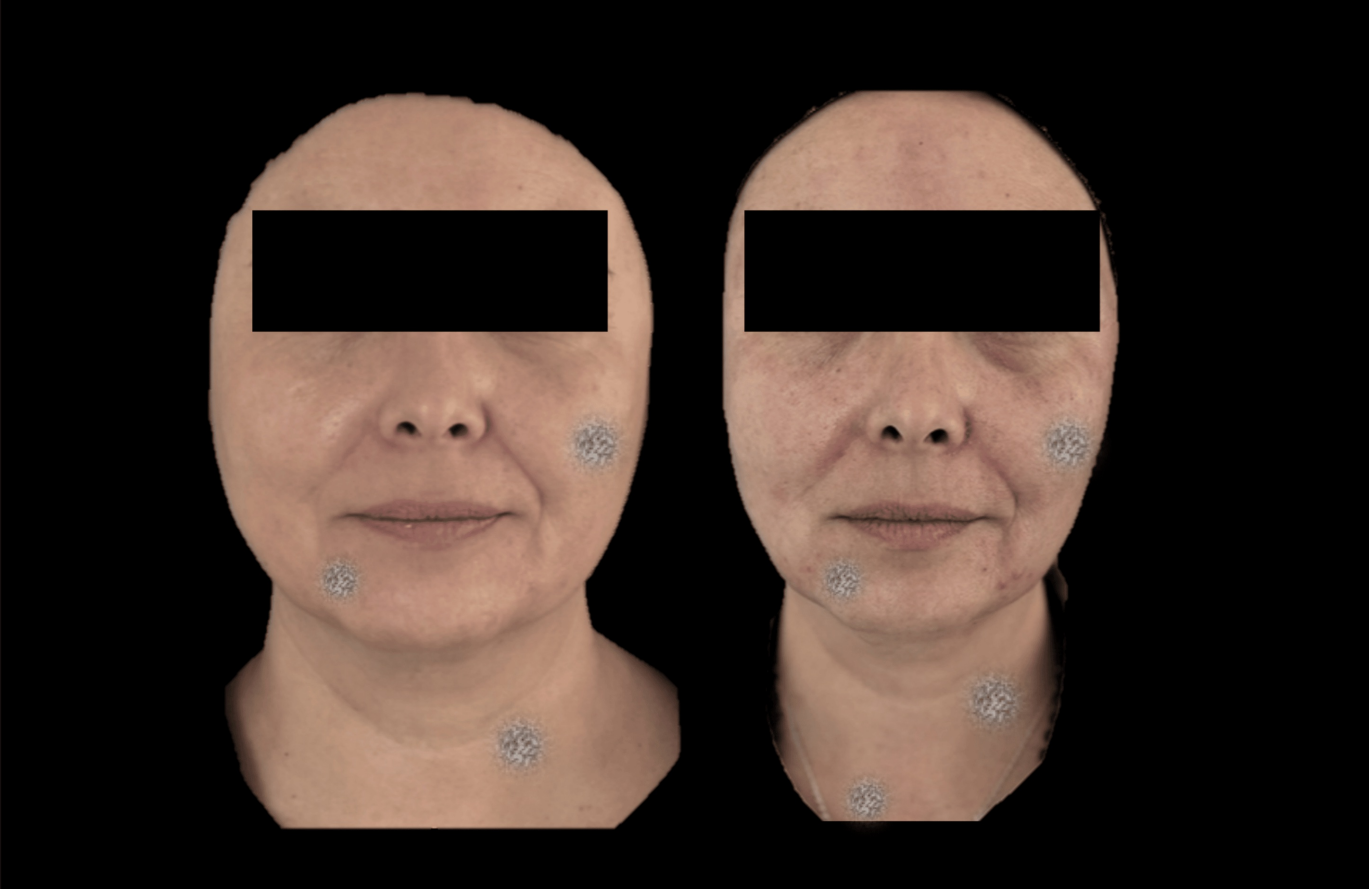
Frontal photograph analyzed using FotoDoc Pro.

**Figure 7:**
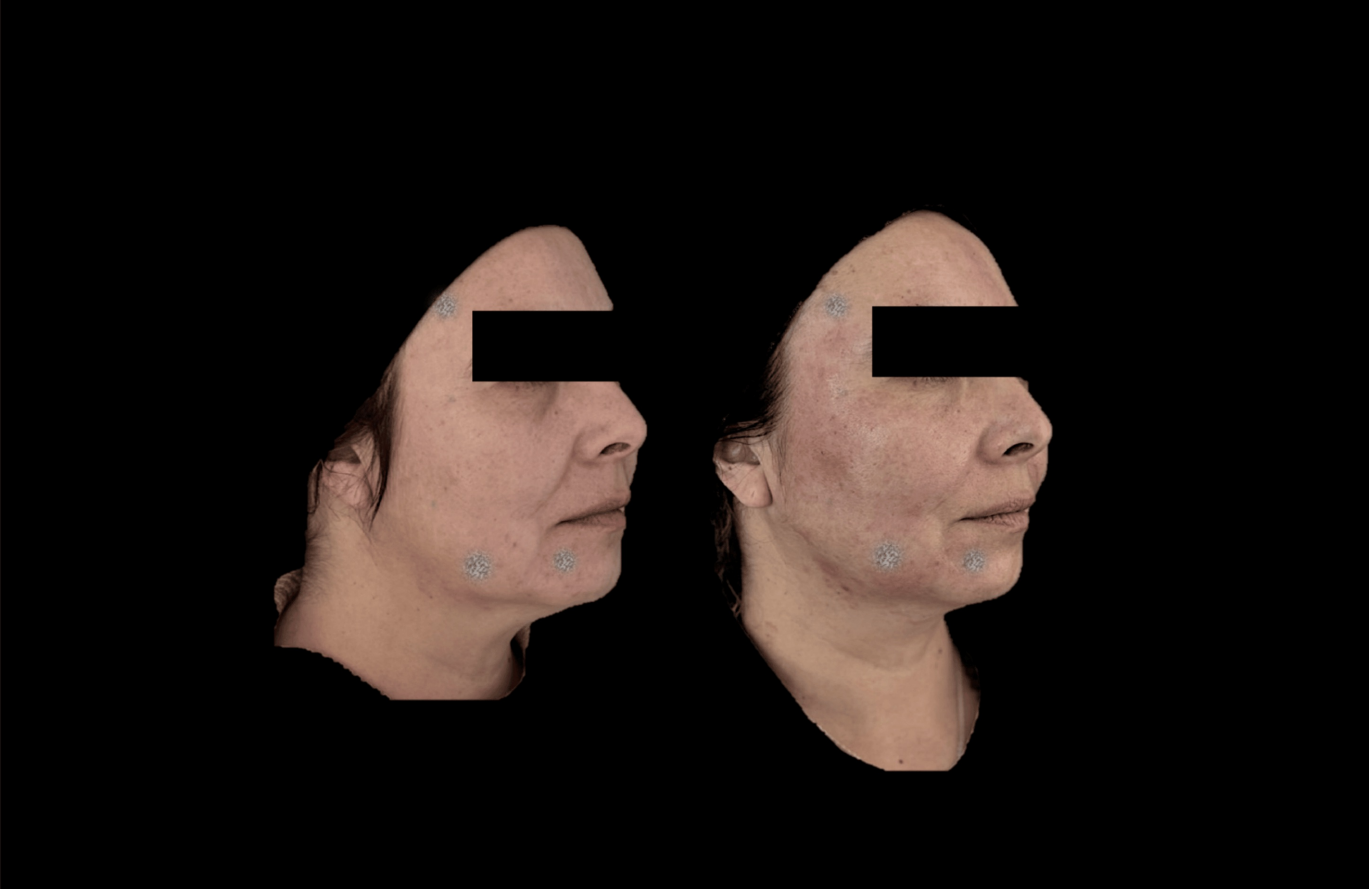
Right lateral photograph analyzed using FotoDoc Pro.

**Figure 8:**
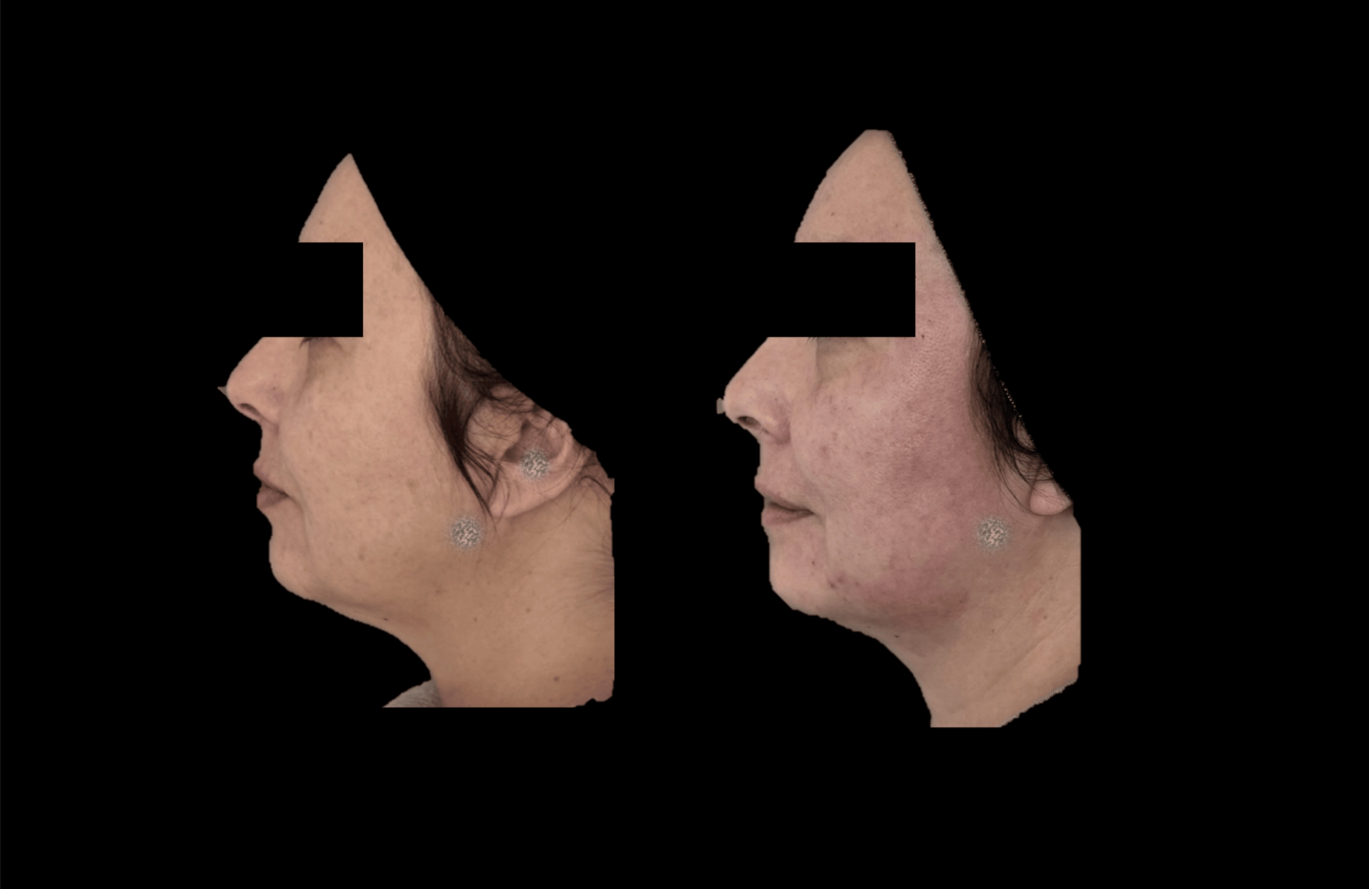
Left lateral photograph analyzed using FotoDoc Pro.

All patients demonstrated aesthetic improvement according to the GAIS scale, with 100% of participants classified within categories 1 to 3. The predominant response was ‘Very Much Improved’ (Category 1), observed in 70% of patients. When combining categories 1 and 2 (‘Very Much Improved’ and ‘Much Improved’), 90% of patients exhibited marked aesthetic enhancement. No patient was classified as ‘No Change’ or ‘Worse.’ Additionally, no adverse events were reported during the follow-up period. Figures 6-8 demonstrate the clinical changes observed in the study, showing clinical improvement.

**Figure 9:**
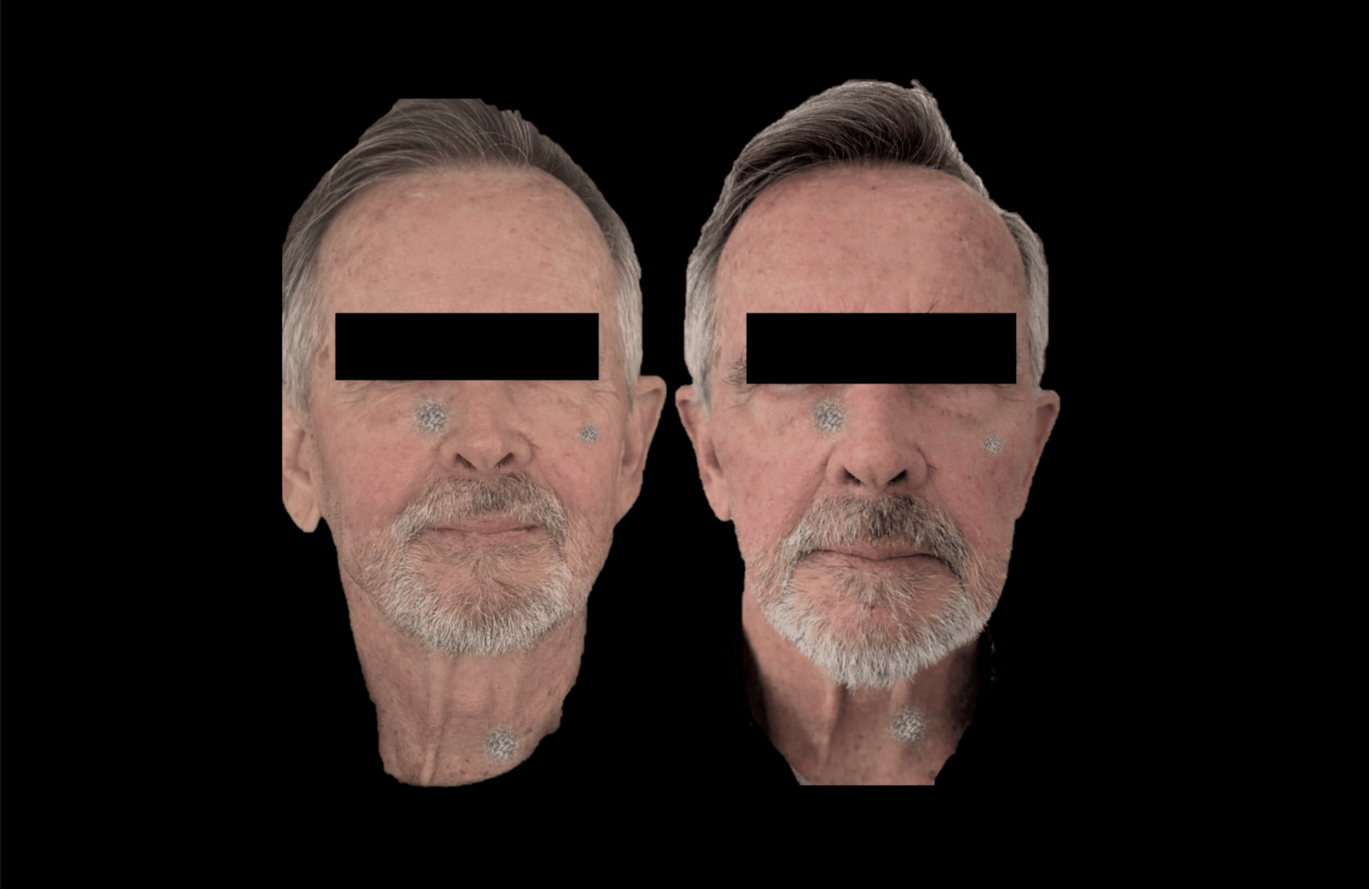
Frontal photograph analyzed using FotoDoc Pro.

**Figure 10:**
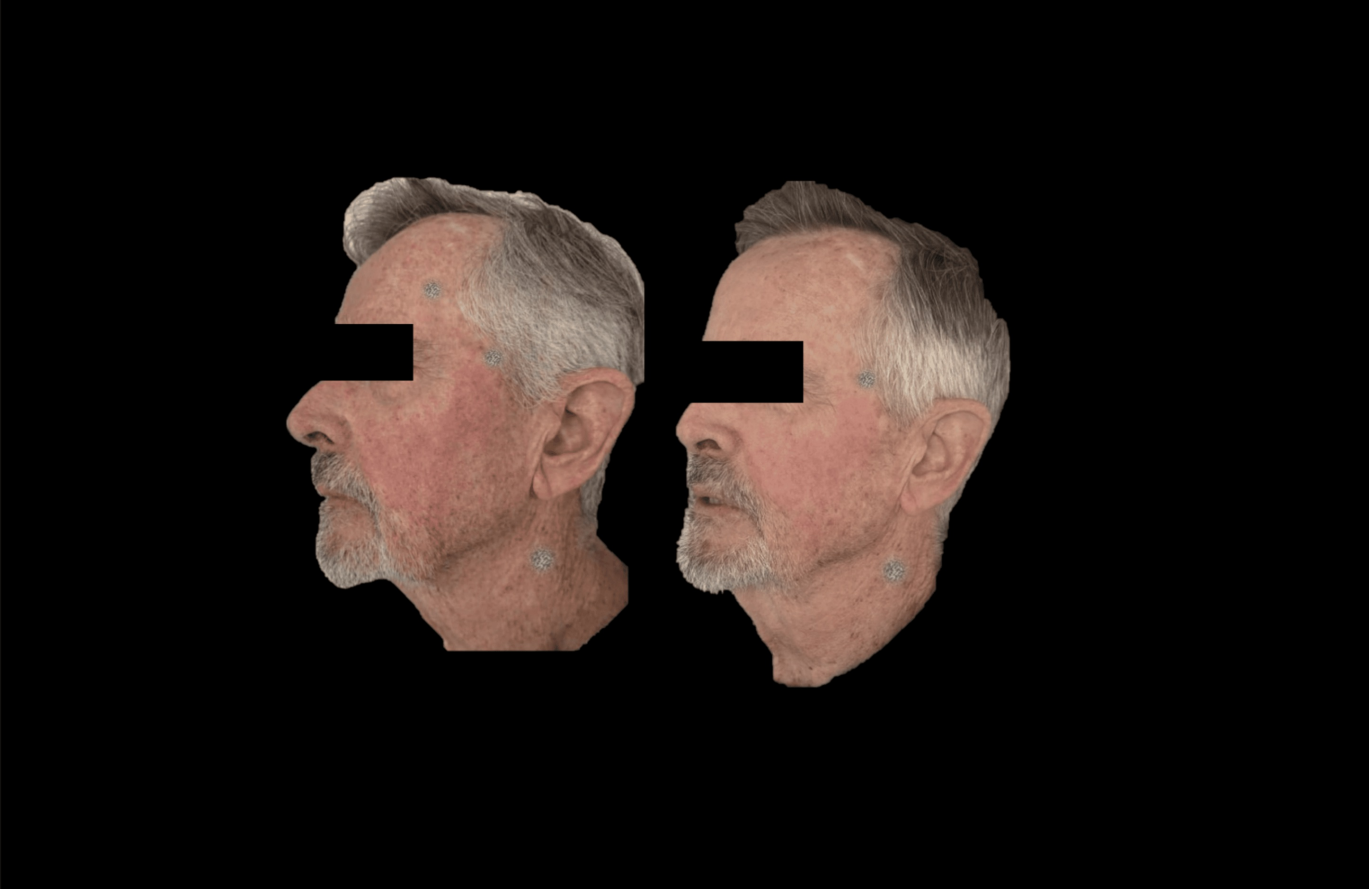
Left lateral photograph analyzed using FotoDoc Pro.

**Figure 11:**
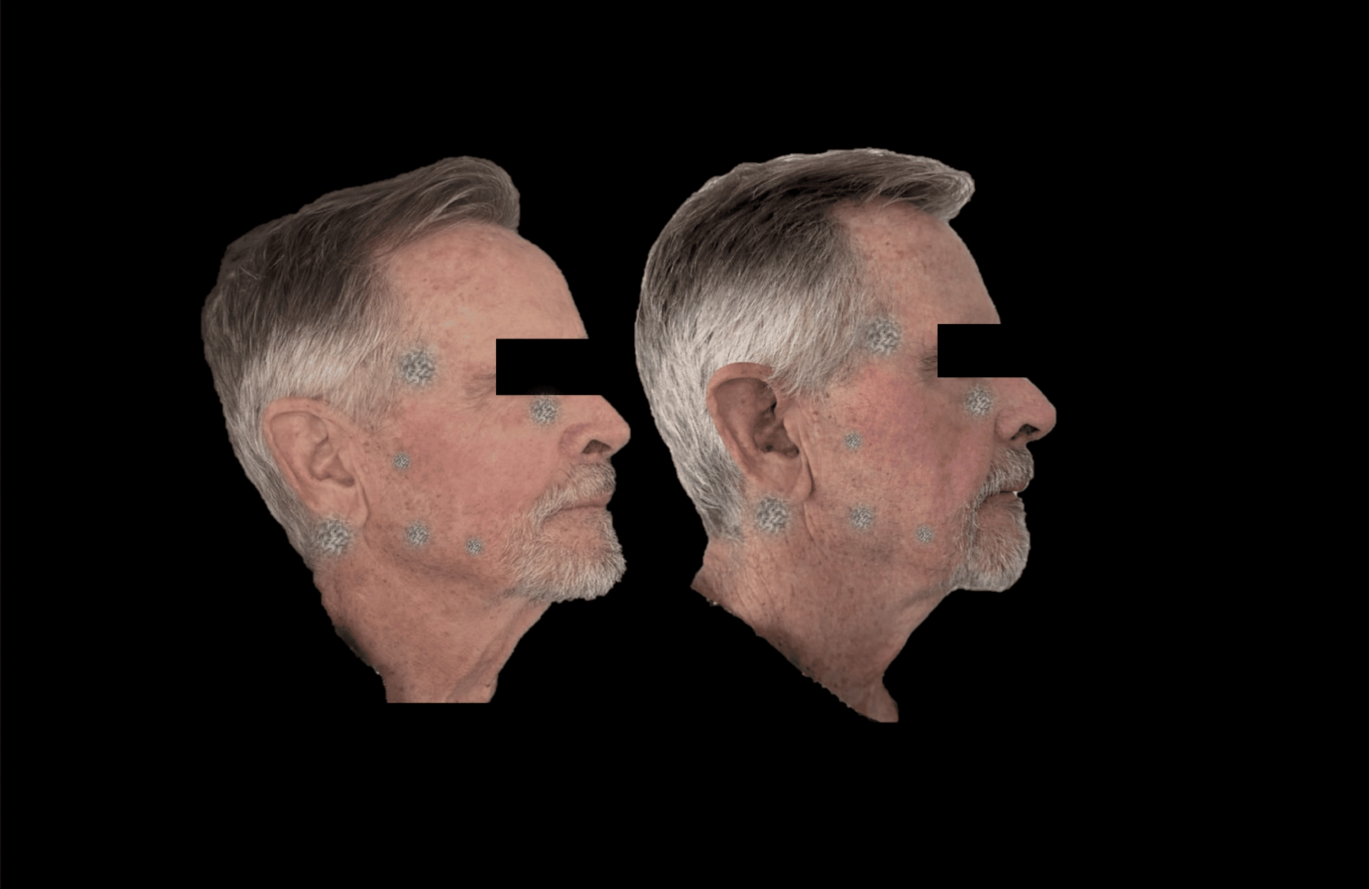
Right lateral photograph analyzed using FotoDoc Pro.

Figures 9-11 present a male patient illustrating the clinical changes consistent with the statistical findings.

**Figure 12:**
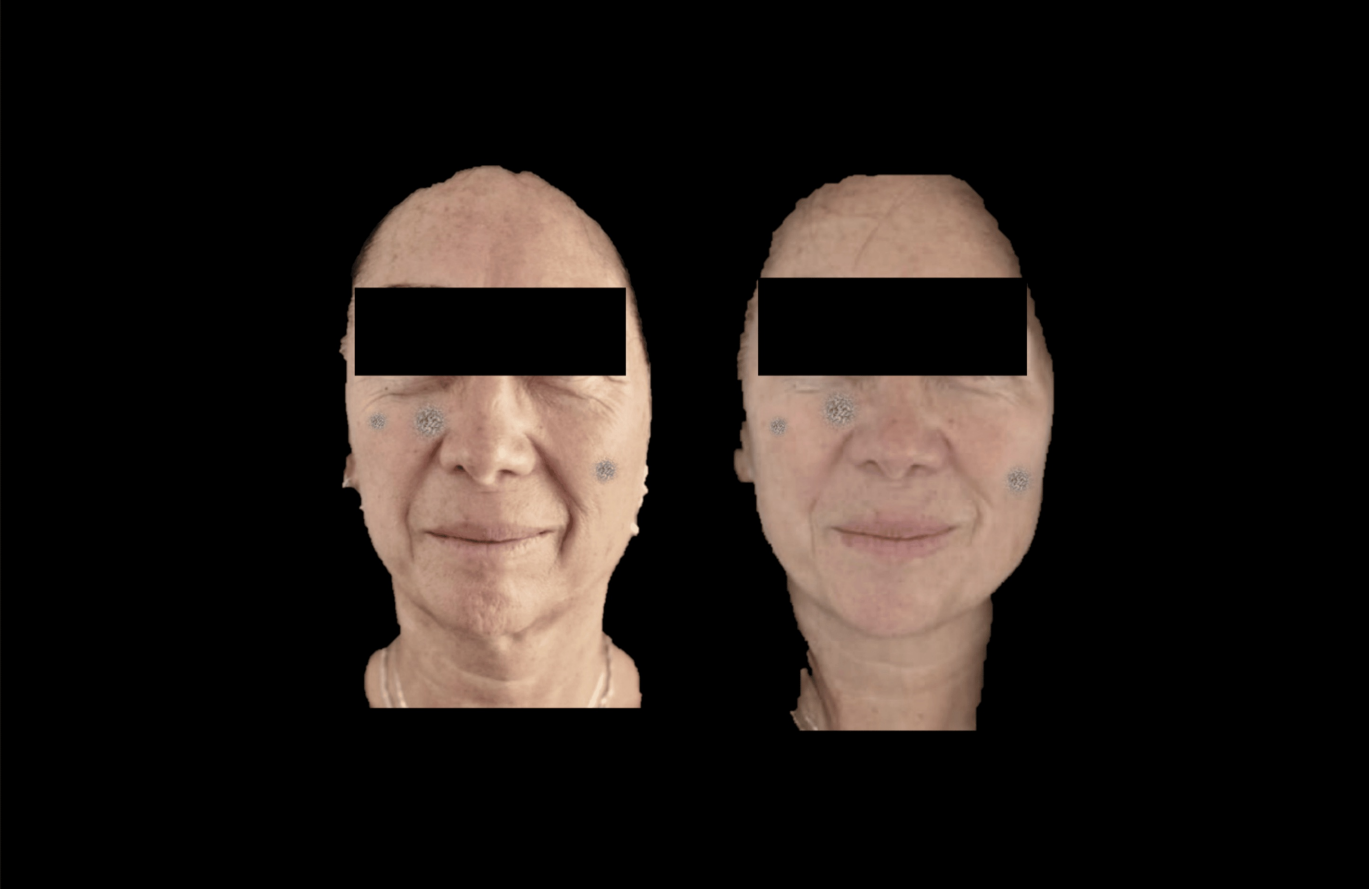
Frontal photograph analyzed using FotoDoc Pro.

**Figure 13:**
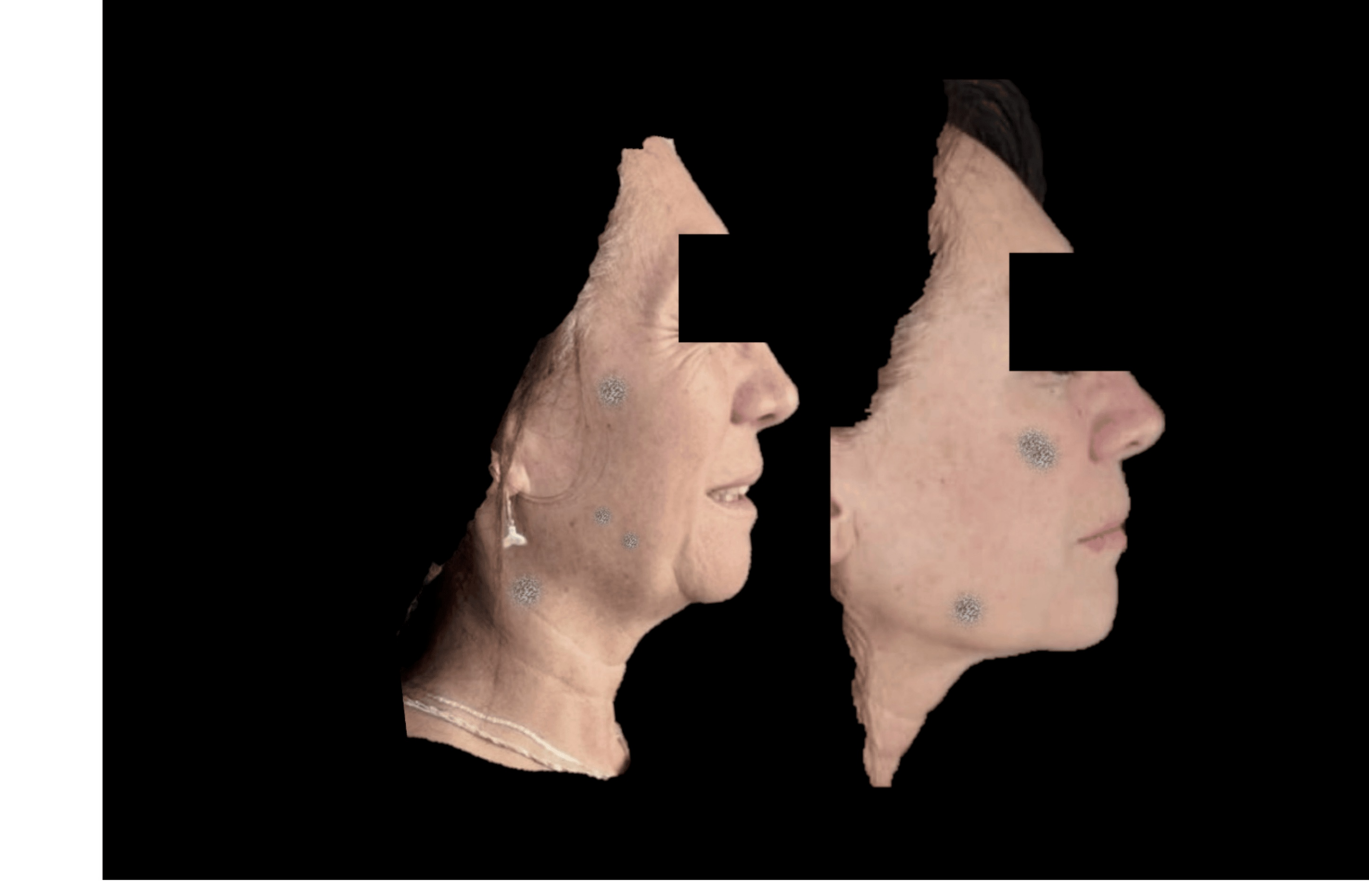
Right lateral photograph analyzed using FotoDoc Pro.

**Figure 14:**
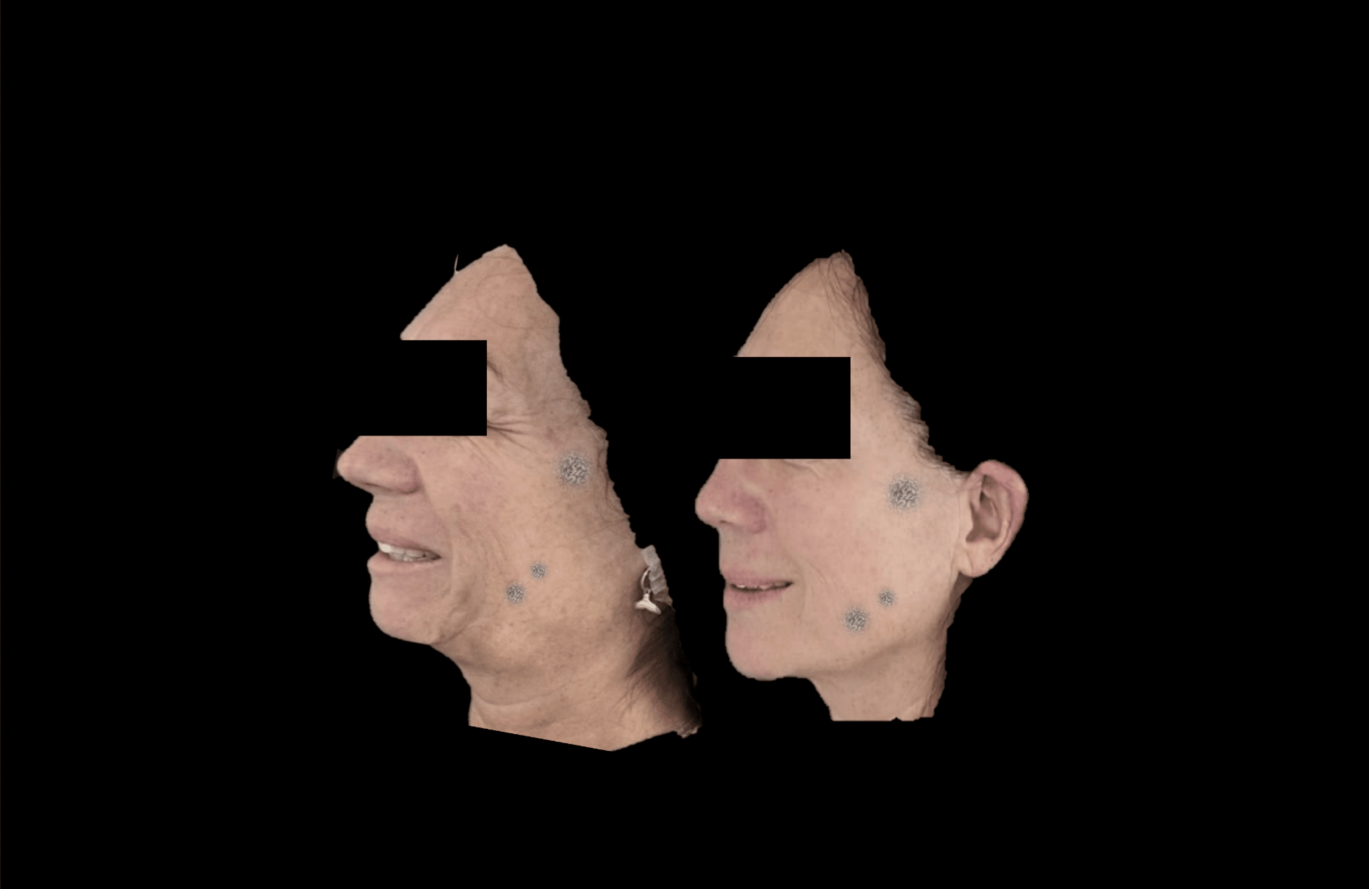
Right lateral photograph analyzed using FotoDoc Pro.

Figures 12-14 present an older female patient illustrating clinical changes consistent with the overall study findings.

## Discussion

Biostimulators have played a fundamental role in addressing the effects of skin aging in patients seeking a facial appearance that aligns with their lifestyle and expectations. In this study, we focused on procedures using Ultracol plus Si, aiming to achieve a synergistic enhancement of Ultracol’s properties. The patients primarily belonged to an older age group and were not seeking volumization, but rather a less fatigued and more harmonious appearance. This population should not be overlooked, as it requires particular attention. The results obtained in this cohort are promising, demonstrating restoration of facial continuity from the malar region to the chin.

Several studies have analyzed Ultracol’s safety profile, its specific characteristics in neocollagenesis, and its minimal adverse effects [8]. Ultracol was selected for this study due to its favorable clinical outcomes, biosafety profile, ease of application, predictable degradation, and controlled durability, which allows for repeated treatment if clinically indicated [10]. These characteristics support its consideration as a safe and effective biostimulator. In our study, the administered dose was 2.5 mL of Ultracol per hemiface, applied in a single session, in contrast to other studies that administered two sessions at four-week intervals [9]. Those studies concluded that Ultracol is an effective alternative for more pronounced age-related skin concerns, such as impaired texture and increased wrinkle depth. In addition, Pedrero TR et al. (2025), in an extensive review, concluded that injectable polydioxanone is an effective and safe biostimulator in aesthetic medicine [10].

The addition of organic silicon was intended to synergistically enhance the regenerative properties of Ultracol. Velazco de Maldonado GJ et al. [11] investigated the release of organic silicon from PDO sutures inserted using the Cartesian technique in patients with signs of facial aging and observed improvements in skin turgor, wrinkle reduction, and tightening of sagging tissue. Histopathological findings revealed that silicon enhances the tissue effects of PDO sutures, leading to increased fibroblast biostimulation and improved collagen fiber quality. Therefore, the incorporation of silicon into Ultracol in our study may further potentiate the known biostimulatory effects of liquid polydioxanone [11]. Kim CM et al. [8] evaluated the biostimulatory effects of Ultracol and PLLA in murine models. Following injections of PLLA and PDO, they measured type I collagen, type III collagen, TGF-β1, TGF-β2, and TGF-β3. Their findings suggest that powdered PDO induces collagen formation more effectively than PLLA, supporting its use as a viable option for collagen stimulation.

In our study, Ultracol was administered using the FA technique, which approximates and repositions fat compartments [11,13]. Louison LD et al. [14] evaluated histological changes using Verhoeff, reticulin, and Masson’s trichrome stains in a patient treated with the FA technique and observed a moderate increase in type I and type III collagen fibers, as well as elastic fibers. They concluded that the FA technique contributes to facial rejuvenation. The rationale for depositing Ultracol within fat compartments is supported by the hypothesis that dermal adipocytes may prolong the effects of biostimulators [15,16]. Furthermore, mechanical stress and other physical factors are believed to stimulate fat-derived stem cells [16]. These findings reinforce the multifunctionality and plasticity of facial dermal adipocytes, as well as their ability to modify their phenotype toward neocollagenesis [17].

This pilot clinical study has limitations, including the absence of a control group and the lack of more objective quantitative assessment tools. Therefore, the findings should be interpreted as preliminary and considered a basis for further research. Nevertheless, the clinical improvements observed were satisfactory. Given the mean patient age of 63.3 years, a group typically presenting a significant burden of age-related changes, this treatment may represent a suitable therapeutic alternative for addressing signs of facial aging. The positive outcomes reported by the blinded evaluator were consistent with the results of the GAIS satisfaction survey.

## Conclusions

This pilot study suggests that the combined use of Ultracol, Si, and the FA technique provides a synergistic approach to facial rejuvenation. This triple synergy integrates the biostimulatory effect of liquid polydioxanone, the structural reorganization of adipose compartments through vector-based adipostructuring, and the regenerative properties of Si. In this predominantly older cohort (mean age: 63.3 years), the treatment was associated with visible improvement in facial contour and skin quality, high patient satisfaction, and an absence of adverse events. These findings support this combined strategy as a safe and effective alternative for addressing age-related facial changes.
